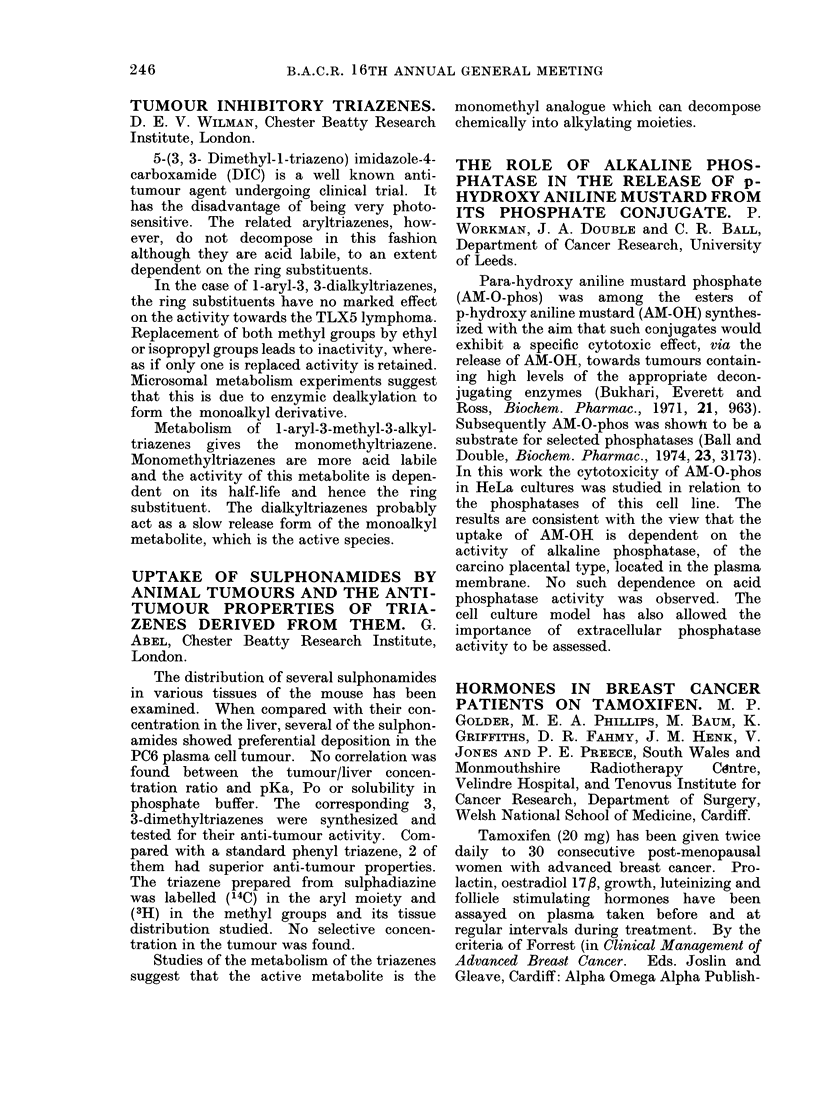# Proceedings: Tumour inhibitory triazenes.

**DOI:** 10.1038/bjc.1975.173

**Published:** 1975-08

**Authors:** D. E. Wilman


**TUMOUR INHIBITORY TRIAZENES.** D. E. V. WILMAN, Chester Beatty Research Institute, London.

5-(3, 3- Dimethyl-1-triazeno) imidazole-4-carboxamide (DIC) is a well known anti-tumour agent undergoing clinical trial. It has the disadvantage of being very photo-sensitive. The related aryltriazenes, however, do not decompose in this fashion although they are acid labile, to an extent dependent on the ring substituents.

In the case of 1-aryl-3, 3-dialkyltriazenes, the ring substituents have no marked effect on the activity towards the TLX5 lymphoma. Replacement of both methyl groups by ethyl or isopropyl groups leads to inactivity, whereas if only one is replaced activity is retained. Microsomal metabolism experiments suggest that this is due to enzymic dealkylation to form the monoalkyl derivative.

Metabolism of 1-aryl-3-methyl-3-alkyl-triazenes gives the monomethyltriazene. Monomethyltriazenes are more acid labile and the activity of this metabolite is dependent on its half-life and hence the ring substituent. The dialkyltriazenes probably act as a slow release form of the monoalkyl metabolite, which is the active species.